# Experimental investigation for nonalcoholic fatty pancreas management using probiotics

**DOI:** 10.1186/s13098-024-01378-w

**Published:** 2024-07-03

**Authors:** Marwa Matboli, Hiba S. Al-Amodi, Shaimaa Hamady, Marwa Ali, Marian MS Roushdy, Amany Helmy Hasanin, Yasmin M. Aboul-Ela, Reda Albadawy, Eman Gomaa, Hala F. M. Kamel, Hind A ELsawi, Laila M. Farid, Mariam B. Abouelkhair, Gena M Elmakromy, Nesma Mohamed Fawzy

**Affiliations:** 1 Medical biochemistry and molecular biology department, Faculty of medicine, Ain Shams University, Cairo, 11566 Egypt; 2 Biochemistry Department, Faculty of Medicine, Umm Al-Qura University, Makkah, 21955 Saudi Arabia; 3 Department of Biochemistry, Faculty of Science, Ain Shams University, Cairo, 11566 Egypt; 4 Clinical pharmacology department, Faculty of medicine, Ain Shams University, Cairo, Egypt; 5 Department of Gastroenterology, Hepatology & Infectious Disease, Faculty of Medicine, Benha University, Benha, 13518 Egypt; 6 Histology and Cell biology department, Faculty of Medicine, Ain Shams University, Giza, Egypt; 7 Department of Internal Medicine, Badr University in Cairo, Badr City, Egypt; 8 Pathology department Faculty of Medicine, Ain Shams University, Giza, Egypt; 9 Endocrinology & Diabetes mellitus unit, Department of Internal Medicine, Badr University in Cairo, Badr City, Egypt

**Keywords:** Nonalcoholic fatty pancreas, Necroptosis, TNF pathway, ZBiotic, *B. subtilis*, obesity

## Abstract

**Background:**

Nonalcoholic fatty pancreatitis (NAFP) presents a pressing challenge within the domain of metabolic disorders, necessitating further exploration to unveil its molecular intricacies and discover effective treatments. Our focus was to delve into the potential therapeutic impact of *ZBiotic*, a specially engineered strain of probiotic *B. subtilis*, in managing NAFP by targeting specific genes linked with necroptosis and the TNF signaling pathway, including TNF, ZBP1, HSPA1B, and MAPK3, along with their upstream epigenetic regulator, miR-5192, identified through bioinformatics.

**Methods:**

Rats were subjected to either a standard or high-fat, high-sucrose diet (HFHS) for eight weeks. Subsequently, they were divided into groups: NAFP model, and two additional groups receiving daily doses of ZBiotic (0.5 ml and 1 ml/kg), and the original *B. subtilis* strain group (1 ml/kg) for four weeks, alongside the HFHS diet.

**Results:**

*ZBiotic* exhibited remarkable efficacy in modulating gene expression, leading to the downregulation of miR-5192 and its target mRNAs (*p* < 0.001). Treatment resulted in the reversal of fibrosis, inflammation, and insulin resistance, evidenced by reductions in body weight, serum amylase, and lipase levels (*p* < 0.001), and decreased percentages of Caspase and Nuclear Factor Kappa-positive cells in pancreatic sections (*p* < 0.01). Notably, high-dose ZBiotic displayed superior efficacy compared to the original *B. subtilis* strain, highlighting its potential in mitigating NAFP progression by regulating pivotal pancreatic genes.

**Conclusion:**

ZBiotic holds promise in curbing NAFP advancement, curbing fibrosis and inflammation while alleviating metabolic and pathological irregularities observed in the NAFP animal model. This impact was intricately linked to the modulation of necroptosis/TNF-mediated pathway-related signatures.

**Supplementary Information:**

The online version contains supplementary material available at 10.1186/s13098-024-01378-w.

## Background

Pancreatic fat infiltration, documented for nearly a century, raises numerous unanswered questions in scientific research [1]. Emerging evidence links pancreatic fat infiltration to various illnesses such as metabolic syndrome, type 2 diabetes, pancreatitis, and pancreatic duct adenocarcinoma (PDAC) [2, 3]. Pancreatic fat accumulation associated with obesity in the absence of substantial alcohol consumption is known as nonalcoholic fatty pancreatitis (NAFP) [4, 5].

There exists a reciprocal relationship between NAFP and nonalcoholic fatty liver disease (NAFLD) as both conditions are linked to fat deposition in the liver and pancreas, respectively [6]. Moreover, fatty pancreas comorbidity has been reported in 50-80% of patients with NAFLD [6, 7].

NAFP pathogenesis is complex process, and like NAFLD is increasingly diagnosed yearly [8]. The metabolic stress resulting from obesity and insulin resistance (IR) in NAFP can cause acute pancreatitis and severe cell damage, which initiate a cascade of signaling pathways and result in acinar cell death and necrosis [9]. The metabolic oxidative stress has an impact on multiple signaling cascades, which in turn induces the tumor necrosis factor (TNF) and nuclear factor kappa beta (NF-kB) signaling pathways to generate proinflammatory cytokines and trigger fibrogenesis [10]. As a result, additional research into these signaling pathways may be helpful in developing new treatments for NAFP.

There are not enough biomarkers currently recognized for the early detection of NAFP. As a result, precise therapeutic targets and non-invasive biomarkers are needed. A small non-coding RNA known as microRNAs (miRNAs) regulates protein-coding gene expression [11]. They are critical controllers of insulin signaling, inflammation, and lipid metabolism [12]. Undoubtedly, bioinformatic analysis makes it easier to determine the relationships between novel applicant RNA species and their use as biomarkers for diagnosis and treatment [13].

Research over the past two decades has highlighted the crucial role of gut dysbiosis in the pathogenesis of numerous diseases, including obesity [14] and diabetes mellitus [15]. Alterations in the diversity, proportions, and dominant species of the gut microbiome are linked to intestinal barrier dysfunction, which can impact the onset and progression of various diseases, including pancreatic disorders [16]. Advances in sequencing methods have revealed the therapeutic potential of interventions targeting gut microbiome composition. Such interventions aim to eliminate harmful taxa or restore beneficial ones, thereby manipulating the host-microbiome community for improved health outcomes [17]. Recent findings indicate that probiotics have anti-inflammatory properties and may reduce the duration of hospital stays for individuals with acute pancreatitis. The mechanisms driving these therapeutic effects likely entail the suppression of the TNF pathway, mitigation of necrosis, and inhibition of adipogenesis [18]. According to [19], ZBiotic is a genetically engineered *B. subtilis* ZB183 that is capable of constitutively expressing the enzyme AcoD, acetaldehyde dehydrogenase. It assists in preventing harmful aldehyde buildup which impacts disease progression by inducing inflammation and oxidative stress [20]. The ZBiotic impact on NAFP, however, is not well demonstrated.

Extensive rodent studies have shown that diets similar to those commonly consumed in modern societies significantly deteriorate various metabolic systems [21]. High-fat and high-sucrose (HFHS) diets lead to excess calorie intake, causing a positive energy balance and obesity. This shift is linked to a rapid decline in whole-body insulin sensitivity, resulting in compensatory hyperinsulinemia and a pre-diabetic state similar to that in humans and is associated with metabolic dysfunction [22].

Considering all of the previously mentioned data, our main goal was to investigate the potential therapeutic benefits of ZBiotic in addressing NAFP by enhancing the inflammatory response and mitigating cell death induced by metabolic stress. Our focus was on analyzing genes associated with the pancreatic necroptosis and TNF signaling pathways (TNF, ZBP1, HSPA1B, and MAPK3), along with their upstream regulator miR-5192, identified through bioinformatics in HFHS-induced NAFP animal model.

## Materials and methods

### Chemicals and drugs

Urethane was obtained from Ralin B.V. (Lijinbaan, The Netherlands). The bacterial culture broth for the original *B. subtilis* strain was procured from Fisher Scientific International Inc. (Cambridge, MA, USA). The engineered form of *B. subtilis*, Zbiotic (ZB183), was acquired as a lyophilized formula (Lot no. 300 L engineering batch ZBT-002a) from The Saskatchewan Research Council (Saskatoon, Canada).

#### Preparation and determination the dosage of the original *B. subtilis* strain and Zbiotic

Previous research on probiotic *B. subtilis* has indicated no safety concerns even at high doses, as demonstrated in studies by [23–25]. Furthermore, when administering lyophilized *B. subtilis* ZB183 spores orally to Wistar rats over a 90-day period, no toxicological effects were observed [20]. Consequently, the dosages for our study were selected based on prior research recommending a dosage of 10^9 CFU/kg, which is considered optimal for most probiotic strains. The original *B. subtilis* strain was cultivated at 30 °C, 220 rpm overnight in LB medium, with or without 5ug/ml chloramphenicol. Following overnight growth, the bacterial culture was diluted tenfold and further cultured at 37 °C, 220 rpm until the optical density at 600 nm (OD600) reached a range between 0.6 and 0.8. Subsequently, the bacteria were preserved in a formulation buffer (comprising KH2PO4, K2HPO4, glycerol, pH 7.5) at a concentration of 10^9^ CFU/ml at -80 °C [26].

For Zbiotic, the target doses were determined and administered based on the colony forming units (CFU) count per weight of the lyophilized spore material. The CFU count of ZBiotic was evaluated using standard serial dilution and plating on LB agar plates. The doses were prepared by suspending lyophilized spores in MilliQ water to achieve a concentration of 20 mg/mL, as outlined by [20], Table 1.

In our present study, two dosages were administered: approximately 1 ml/kg (equivalent to 20 mg Zbiotic/mL) and 0.5 ml/kg (equivalent to 10 mg Zbiotic/0.5mL). The volume of dose administered was calculated for each individual animal on the first day of treatment and adjusted based on subsequent body weight measurements recorded at various intervals throughout the study.

**Table 1:** Details of CFU/g and doses administered

Count of Lyophilized Zbiotic	Desired Count of Lyophilized Zbiotic	Calculated Dose	Dose volume
≈ 52 × 10^^9^ CFU/g	10^^9^ CFU/kg/day	≈ 20 mg/kg/day	-1 ml/kg (ZB-1 ml group) -0.5 ml/kg (ZB-0.5 ml group)

### Experimental animals and design

Male Wistar rats, weighing between 150 and 170 g, were purchased from Nile Pharmaceuticals Company in Cairo, Egypt, and kept in a temperature-controlled environment (20 ± 2 ◦C) with a 12-hour light/dark cycle. They also had unlimited access to water and standard rat chow. The Banha University Faculty of Medicine Research Ethics Committee approved the experimental protocol (Ethical Approval Number: MoHP0018122017, 1017), and the experimental research was carried out in compliance with the Declaration of Helsinki’s guidelines (Figure S1).

Non-alcoholic fatty pancreas model (NAFP) was created by giving the rats a diet high in fat and sugar (HFHS) [27], according to Table 2. An acclimatization period of one-week was allowed after which six groups of rats (*n* = 8 for each group) were randomly assigned; the normal control group (given normal chow diet), NAFP model group (given a HFHS diet), HFHS + MilliQ group (given a HFHS diet and received 1 ml/kg/day of MilliQ water), ZB-0.5 group (given a HFHS diet and received 0.5 ml/kg/day of Zbiotic), ZB-1 group (given a HFHS diet and received 1 ml/kg/day of Zbiotic) and *B. subtilis* group (given a HFHS diet and received 1 ml/kg/day of original *B. subtilis* strain) as a control group [20]. The experimental protocol was executed over 12 weeks where rats were fed either normal chow or HFHS diet and in the final 4 weeks the treated groups received daily Zbiotic by oral gavage, alongside the ongoing diet regimen. The HFHS diet continued throughout the Zbiotic treatment period.

**Table 2:** Ingredients of the Standard chow and high-fat and high-sucrose (HFHS) diets

Diet Composition (gm)	HFHS Diet	Standard Chow
Casein	160.0	140.0
L-cysteine	1.8	1.8
Corn starch	220.7	620.7
Sucrose	300.0	100.0
Fiber	50.0	50.0
Soybean oil	40	40.0
Lard	180.0	0
Vitamin & mineral mix	45	45
Choline	2.5	2.5

### Blood sampling and pancreas tissue collection

Body weight was recorded daily for all rats. Rats were given an IP injection of a single dose of urethane (1.2 g/kg) to induce anesthesia at the end of the 12th week [28] prior to scarification. The retroorbital vein was used to extract blood samples in order to separate serum followed by storage at − 20 °C for additional biochemical analysis. Pancreas tissue was dissected and weighed, and part of it was immediately kept at -80 °C for protein and RNA tests, but the other sections were promptly preserved in 10% neutral buffered formaldehyde for examination using histology and immunohistochemistry.

### Serum biochemical analysis

#### Fasting serum glucose and glycated hemoglobin (HbA1C)

The multifunctional biochemistry analyzer (AU680, Beckman Coulter Inc., Brea, CA, USA) was used to quantitatively measure fasting serum glucose and glycated hemoglobin (HbA1C). The multifunctional biochemistry analyzer required approximately 100 µL of serum samples.

#### Nonalcoholic fatty pancreas (NAFP)model markers

According to the manufacturer’s recommendations, serum insulin was tested using a rat sandwich ELISA kit from Invitrogen (Cat. NO. ERINSX10, Waltham, Massachusetts, USA). Using commercial kits purchased from Erba Diagnostics (Miami, Florida, USA), serum lipase and amylase were tested in accordance with the instructions provided with each assay. The volumes of serum utilized for insulin, lipase, and amylase assays were determined in accordance with the manufacturer’s protocols, with volumes of 50 µL, 10 µL, and 25 µL, respectively. The following formula was used to determine the homeostasis model assessment-insulin resistance (HOMA-IR) = [fasting serum glucose (mg%) x fasting serum insulin (µU/ml)]/405 [29].

### Pancreatic immunohistochemistry assays and histological examination

#### Preparation of tissues

Rats underwent a fasting period lasting 8 h before the rapid dissection of the pancreas through an abdominal incision. The dissected pancreatic samples were then fixed in 10% formol saline. Following fixation, the samples underwent dehydration using a series of increasing alcohol concentrations, clearing with methyl benzoate, and embedding in paraffin blocks. Sections, with a thickness of 5 μm, were cut and subjected to staining with hematoxylin and eosin (H&E) as well as Masson’s trichrome stain for collagen fiber identification. Additional sections of paraffin, arranged on positively charged slides, underwent an immune reaction for caspase 3. Positive caspase 3 immune-histochemical reactions manifested as a brown nuclear and cytoplasmic response. A ready-to-use Caspase-3 kit was employed for apoptosis detection. Negative controls followed the same protocol, excluding the primary antibody. A positive control, using a liver section, was included in the procedure. The Lab Vision kit from CA, USA, was utilized for these experiments. Mayer’s hematoxylin was employed for the counterstaining of the slides. Positive controls were processed in accordance with the same protocol outlined by Suvarna et al. (2018) [30]. Moreover, more Pancreatic sections were placed on positively charged slides, underwent an immune reaction for nuclear factor kappa Negative controls were conducted in the absence of primary antibodies. A positive control, using testis section, was included in the procedure. The Lab Vision kit from Santa Cruz Biotechnology, Santa Cruz, CA, USA, was utilized for these experiments. Cells showing staining in both the cytoplasm and nucleus were considered positive. also, Positive controls were treated following the identical protocol as outlined by Suvarna et al. (2018) [30].

A morphometric and statistical analysis was conducted using the Leica Q win V.3 image analyzer program installed on a computer in the Department of Histology and Cell Biology, Faculty of Medicine, Ain Shams University. The computer was linked to a Leica DM2500 microscope located in Wetzlar, Germany. Pancreatic slides from all experimental groups underwent morphometric examination. Measurements were taken from five distinct non-overlapping field from slide derived from every animal, (i.e., five randomly chosen, non-overlapping fields studied on every slide). The subsequent parameters were measured as follows ; a; the mean area percentage of collagen fibers in Masson’s trichrome stained sections at an objective lens magnification of X20, b; the mean area percentage of positive caspase-3 reaction sections at X20 magnification, and c; The mean area percentage of nuclear factor kappa reaction sections at X20 magnification.

### Bioinformatics Set up

Herein to identify RNA signatures that may contribute to pathogenic inflammatory and cell death processes of nonalcoholic fatty pancreas, we have analyzed several microarray databases. Our search focused on RNA species, including mRNA and miRNAs, which are highly involved in necroptosis and the upstream regulating Tumor Necrosis Factor (TNF)-mediated signaling pathway.

### Selection of the candidate mRNA species

#### Data resources and analysis of differentially expressed (DEGs)

The gene chip datasets containing expression profiling by high throughput sequencing for pancreatic injury were obtained through the Gene Expression Omnibus (GEO) database (https://www.ncbi.nlm.nih.gov/geo/, accessed in Jan 2023) [31]. The search was restricted to homo sapiens and experimental studies that compared the RNA expression in pancreatitis patients with healthy controls. As a result, GSE194331 [32] was selected as it contained samples from 87 pancreatitis patients with varying levels of severity (Mild = 57, Moderately Severe = 20, Severe = 10) and from 32 healthy controls. The annotation information of Gene Chip was acquired using the GPL16791 Illumina HiSeq 2500 platform.

Then, the microarray data from dataset GSE194331 was analyzed using the online database tool GEO2R (https://www.ncbi.nlm.nih.gov/geo/geo2r/, accessed in January 2023) to detect genes with differential expressions. The criteria for screening used in this study were FDR < 0.05 and adj p value < 0.05. Probes without identified gene symbols were removed, and all DEGs were then displayed through volcano graphs for better visualization.

#### Enrichment analyses of DEGs

2013 Enrichr was used to perform KEGG pathway and biological gene ontology enrichment analyses [33] (https://maayanlab.cloud/Enrichr/, accessed in January 2023) to identify the biological processes and biological pathways significantly enriched in the DEGs. The DEGs then were filtered based on the terms associated with necroptosis and the upstream regulating TNF-mediated signaling pathway.

#### Protein–protein network (PPI) analysis

The filtered necroptosis/TNF-mediated signaling pathway-related After that, DEGs were uploaded to the STRING database (https://string-db.org/, version 12, accessed in January 2023) to build networks of protein-protein interactions (PPIs). We used Cytoscape software (version 3.7.2) to visualize the PPI networks, which were constructed using interactions with a combined score of > 0.15. Finally, we analyzed the obtained networks using the CentiScaPe app [34] to determine the top hundred hub genes. The degree of each node was calculated, and we defined genes with a degree > 25 as hub genes.

#### Selection of candidate genes

The study identified two candidate genes, namely Z-DNA Binding Protein 1 (ZBP1) and Heat Shock Protein Family A-Member 1B (HSPA1B), from the hundred hub genes, as well as two additional genes, TNF and MAPK3, which were found to be strongly interacted with the two selected hub genes and highly related to pancreatic function-regulating signaling pathways. The STRING database for the PPI network was then utilized to analyze the four genes that were chosen.

### Selection of the candidate miRNA species

To retrieve the upstream epigenetic miRNAs interacting with the four chosen mRNAs, miRWalk 3.0 (http://mirwalk.umm.uni-heidelberg.de/*)* was utilized on Jan 2023. To visualize the enrichment of the selected miRNA, DIANA tools: v.3 of mirPath was utilized (http://www.microrna.gr/miRPathv3*).*

#### Total RNA extraction and quantitative polymerase chain reaction (qPCR)

Using a miRNEasy extraction kit (Qiagen, Hilden, Germany, Cat. No. 217,004) and the provided procedure, total RNA extraction, including mRNAs and miRNAs, was carried out on 60 mg of frozen pancreas tissue samples. Total RNA concentration and purity were measured using NanoDrop (Thermo Scientific, USA), and the purity of the isolated RNAs was set to 1.8–2 (A260/A280).

The miScript II RT (Cat. No. 218,161, Qiagen, Germany) was used to reverse-transcribe the pancreas tissues’ RNA into complementary DNA. Using a QuantiTect SYBR Green Master Mix Kit (Qiagen, Germany, Cat. No. 204,143) for ZBP1, MAPK3, HSPA1B, and TNF mRNAs and a miScript SYBR Green PCR Kit (Qiagen, Germany, Cat. No. 218,073) for miR-5192, the relative expression of the chosen RNA species in the pancreatic tissue samples was measured.

On using 7500 Fast System (Applied Biosystems, Foster City, California), real-time (RT)-qPCR was carried out. As housekeeping genes, SNORD72 and GAPDH have been used. Using the formula RQ = 2 ^-^^ΔΔCt^, RNA expression’s relative quantification was computed [35].

#### Statistical analysis

All values were presented as mean ± standard deviation. Statistical differences among the groups were analyzed using one-way analysis of variance (ANOVA), and post-hoc tests were conducted using SPSS program version 19 (IBM Corporation, Somers, New York, USA). Results were considered statistically significant if the P-value was less than 0.05.

## Results

### HFHS feeding and zbiotic impact the weight of the body and food intake

**Fig. 1:**
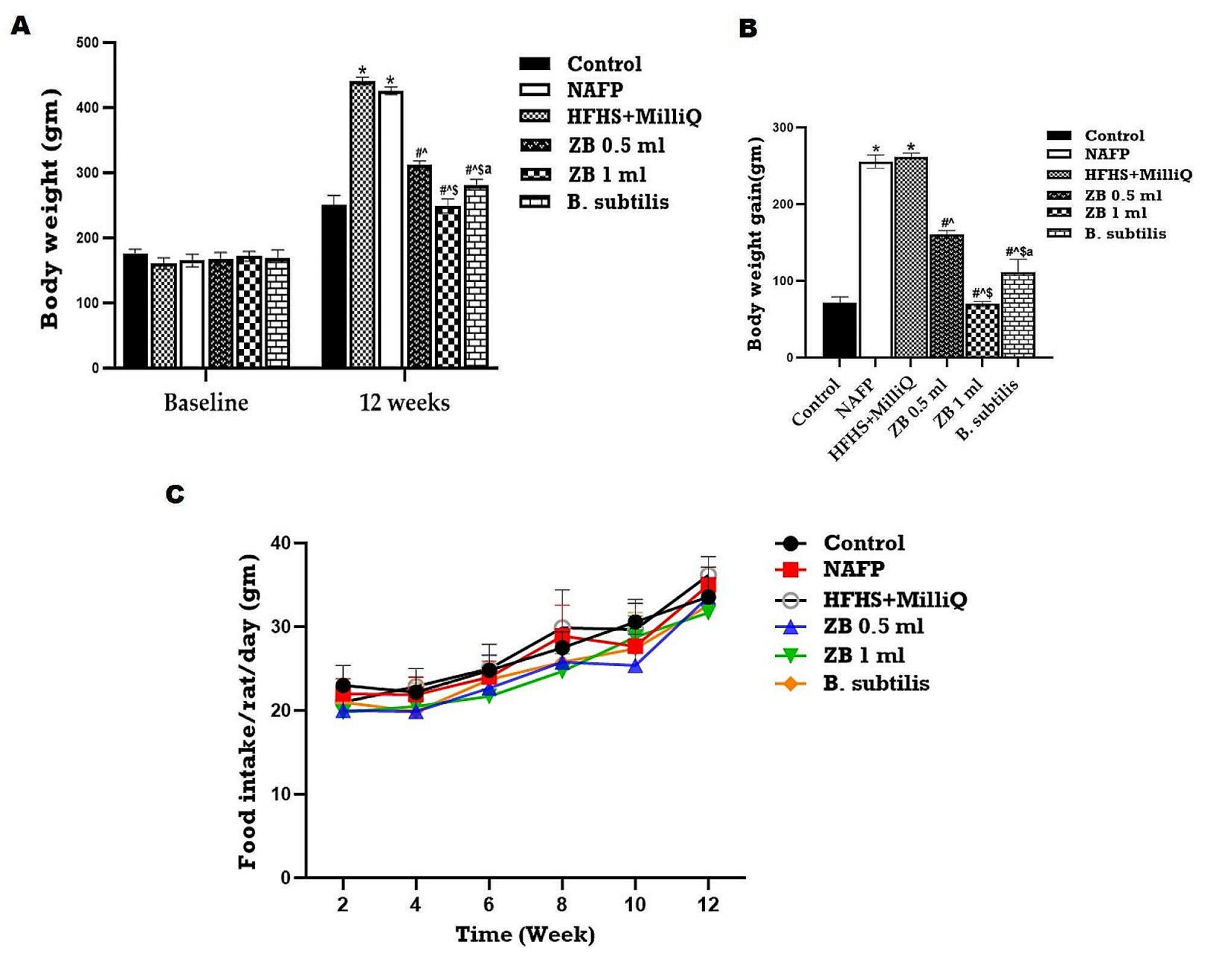
Impact of ZBiotic treatment on the (**A**) Body weight, (**B**) Body weight gain & (**c**) Food intake. One-way analysis of variance and Tukey’s test yielded a mean ± SD for the data. *n* = 8 rats per group, ^*^p < 0.001 in relation to control, ^**#**^*p* < 0.001 in relation to NAFP group, ^**^**^*p* < 0.05 in relation to HFHS + MilliQ group ^**$**^*p* < 0.05 in relation to ZB 0.5 ml group and ^**a**^*p*<0.05 in relation to ZB 1 ml group

In comparison to the control animals, the experimental HFHS-fed rats and HFHS + MilliQ rats (Table S1, Fig. 1) revealed significant rise in body weight (*P* < 0.001). There was no significant change between the NAFP and HFHS + MilliQ group. Conversely, following a 4-week ZBiotic treatment, the body weight of HFHS rats (ZB-0.5 & 1 ml) was significantly lower than that of model rats. Regarding the average food intake, upon ZBiotic treatment, the diet intake among the groups did not differ significantly and the animals did not reduce their feeding. The original *B. subtilis* strain had a similar ameliorative pattern to ZB-05 ml group. The findings showed that Zbiotic had an improving effect on the further body weight gain observed in the untreated FP group.

### Impact of ZBiotic therapy on the blood biochemical indices

In contrast with the model and the HFHS + MilliQ groups, two treated animal groups showed dose-dependent improvements in all of the investigated mentioned biochemical variables including serum glucose, insulin, and insulin resistance. In comparison to the model group, ZBiotic treatment also reduced serum amylase and lipase levels. This beneficial effect was more pronounced in the high dose ZBiotic group (Table S2). In addition, there were significant differences between the original *B. subtilis* group and ZB-1 ml group in the investigated parameters.

### Histological observations

**Fig. 2:**
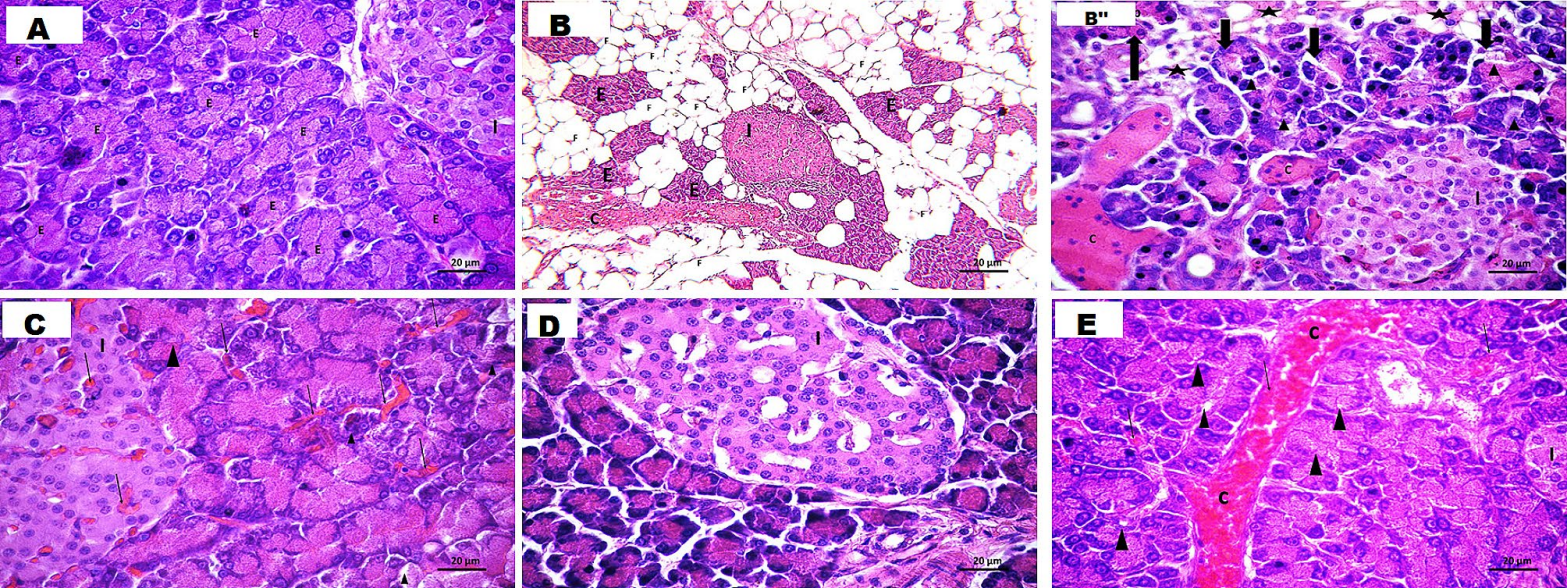
In the (H&E) stained pancreas sections of A: Control group, the exocrine pancreatic acini **(E)** appear densely arranged, displaying basal basophilia and apical acidophilia, the acinar cells exhibit nuclei in an open phase at the basal end. the endocrine part in the form of the islet of Langerhans **(I)** is evident, it is formed of branching and anastomosing cord of acidophilic cell with vesicular nucleus. The cells are separated by spaces of blood capillaries, B& B’: NAFP model group exhibits a disruption in the pancreatic architecture, characterized by the loss of normal structure. Notably, fat deposition **(F)** is observed among exocrine pancreatic acini **(E)** and endocrine islet of Langerhans **(I)**. it reveals a congested and dilated blood vessel **(C)**, This photomicrograph reveals the presence of congested dilated blood vessel **(C)**, surrounded by pancreatic acini showing distortion, with certain acinar cells presenting cytoplasmic vacuolation and pyknotic nuclei (▲). Additionally, distinctive clear spaces were evident between the acini (*) showing mononuclear cellular infiltration. The endocrine islet of Langerhans **(I)** shows many congested, C: ZB 0.5 ml group shows pancreatic lobules with tightly packed acini, focal regions exhibited structural alterations, with some acini displaying pale vacuolated cytoplasm. Both the exocrine pancreatic acini and islet of Langerhans are surrounded by congested dilated blood vessels **(C)**, D : ZB 1 ml group, it reveals that most of pancreatic acini are densely packed exhibited numerous zymogen granules. The nuclei appeared basal and vesicular.as regarding the islet of Langerhans **(I)** it showed cords of cells with vesicular nuclei. These cords are branching and anastomosing surrounding blood capillaries. E: *B. subtilis* group, shows pancreatic lobules with tightly packed acini, focal regions exhibited structural alterations, with some acini displaying pale vacuolated cytoplasm ( ▲ ). Both the exocrine pancreatic acini and islet of Langerhans are surrounded by congested dilated blood vessels. Moreover, there is congested dilated blood vessel within lobule (C)

The potential improving effects of Zbiotic on the inflammatory and fibrotic changes of pancreatic tissues have been evaluated using H&E and Masson trichrome staining. Following microscopic analysis of the pancreatic H&E sections in the control group, the exocrine pancreatic acini (E) demonstrate tightly packed acini, characterized by basal basophilia and apical acidophilia. The cells called acinar reveal nuclei in an open phase at the basal end. The endocrine component was represented by the islet of Langerhans (I), it was clearly visible. It consists of branching and anastomosing cords of acidophilic cells with vesicular nuclei. These cells are separated by spaces containing blood capillaries (Fig. 2).

As regards the model group, an absence of regular lobulation was evident. Fat cells replace the large sections of the pancreatic parenchyma, i.e., areas of fat necrosis that are evident. The intact acini were interspersed within fat, but residual acini exhibited distortion and vacuolation. Intense mononuclear cellular infiltration was present, and the connective tissues between and within the lobules appeared thicker. The endocrine islet of Langerhans (I) was easily noticed. it showed many congested blood vessels, some nuclei appear pyknotic at the periphery of the islet (Fig. 2).

In ZB 0.5 group, it was found that pancreatic lobules showed tightly packed acini, and certain focal regions display structural alterations, notably with some acini featuring pale vacuolated cytoplasm. Both the exocrine pancreatic acini and the islet of Langerhans were encircled by congested and dilated blood vessels(Fig. 2).

However, in the ZB 1 group, the pancreas displayed a thin interlobular septa and densely packed pancreatic acini characterize the normal structure. The majority of acini were made up of regular acinar cells with vesicular nuclei, apical secretory granules, and basal basophilia. Concerning the islet of Langerhans (I), it revealed cords of cells with vesicular nuclei. These cords exhibited a branching and anastomosing pattern around the surrounding blood capillaries. it resembled a healthier state compared to the other experimental groups (Fig. 2). The observed pathological features in the *B. subtilis* group closely resembled those found in the ZB-0.5 ml group.

**Fig. 3:**
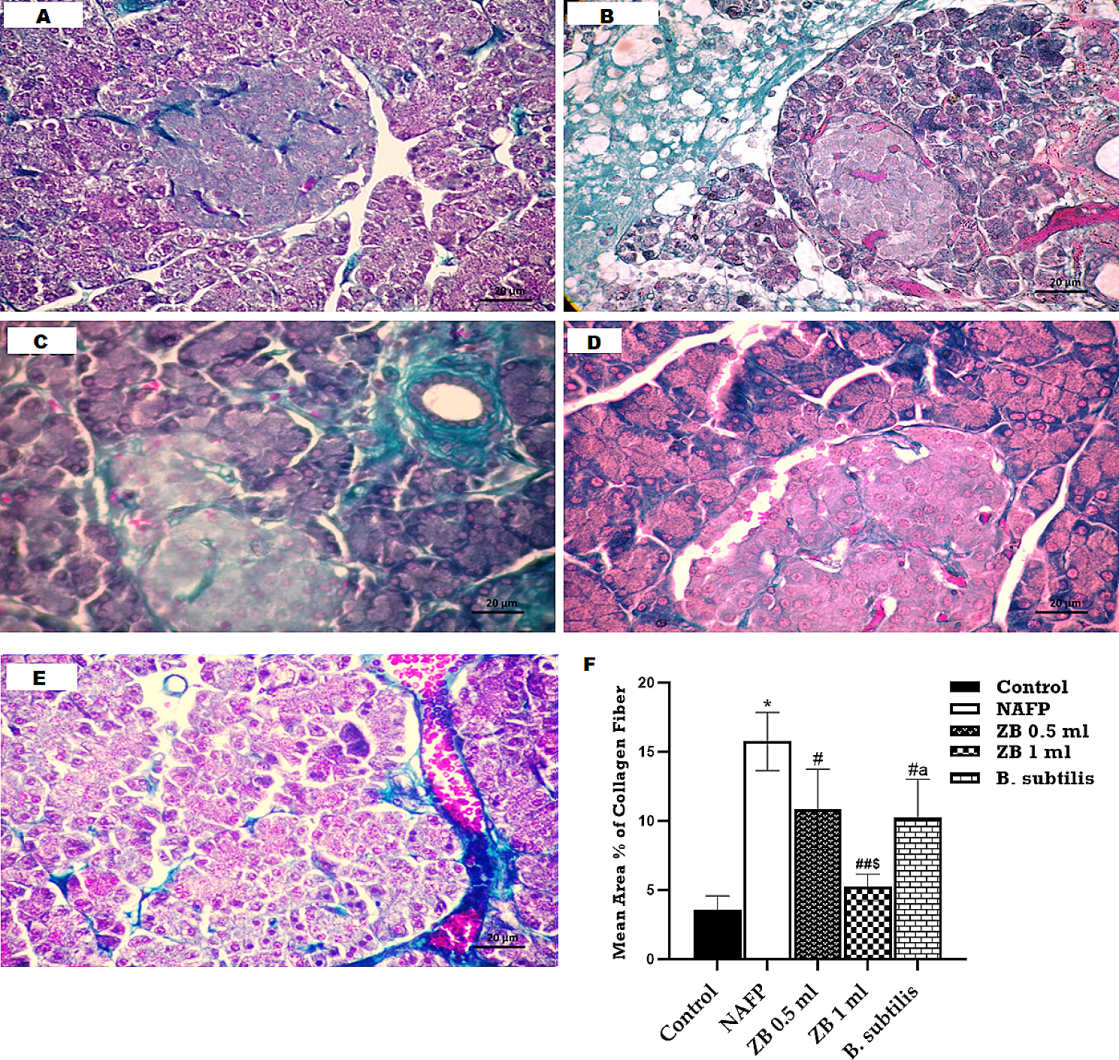
Masson trichrome- stained pancreas sections of A: Control group illustrates a faint green coloration of collagen fibers dispersed among the closely packed pancreatic acini and islets of Langerhans, B: NAFP model group exhibits a significant deposition of green-colored collagen fibers in the interlobular septa, interspersed among the disrupted acini and among endocrine islet of Langerhans, C: In ZB 0.5 ml group, collagen fibers are notably evident, particularly surrounding blood vessels and distorted acini and surrounding islets of Langerhans, D: ZB 1 ml group, there is mild deposition of collagen fibers surrounding the acini and islet of Langerhans. E: *B. subtilis* group, collagen fibers are present, surrounding blood vessels and distorted acini and islets of Langerhans. **F**: morphometric and statistical analysis of the area percentage of collagen. One-way analysis of variance and Tukey’s test yielded a mean ± SD for the data. *n* = 6 rats per group^*^p < 0.01 in relation to control, ^##^*p* < 0.001 & ^#^*p* < 0.05 in relation to NAFP group, ^$^*p* < 0.05 in relation to ZB 0.5 ml group and ^a^*p*<0.05 in relation to ZB 1 ml group

In Masson’s trichrome-stained sections, there was a progressive rise in the deposition of collagen fibers observed in model, ZB 0.5 ml and *B. subtilis* groups, reaching a maximum in model group. It showed a non-significant increase of collagen deposition in ZB 1 group as compared to the control, collagen fibers were still evident around blood vessels distorted pancreatic acini, distorted acini, and thickened interlobular septa. The amount of collagen fibers in these treated groups appeared to be less than that observed in the model group. These findings were further supported by the results of the statistical study (Fig. 3).

**Fig. 4:**
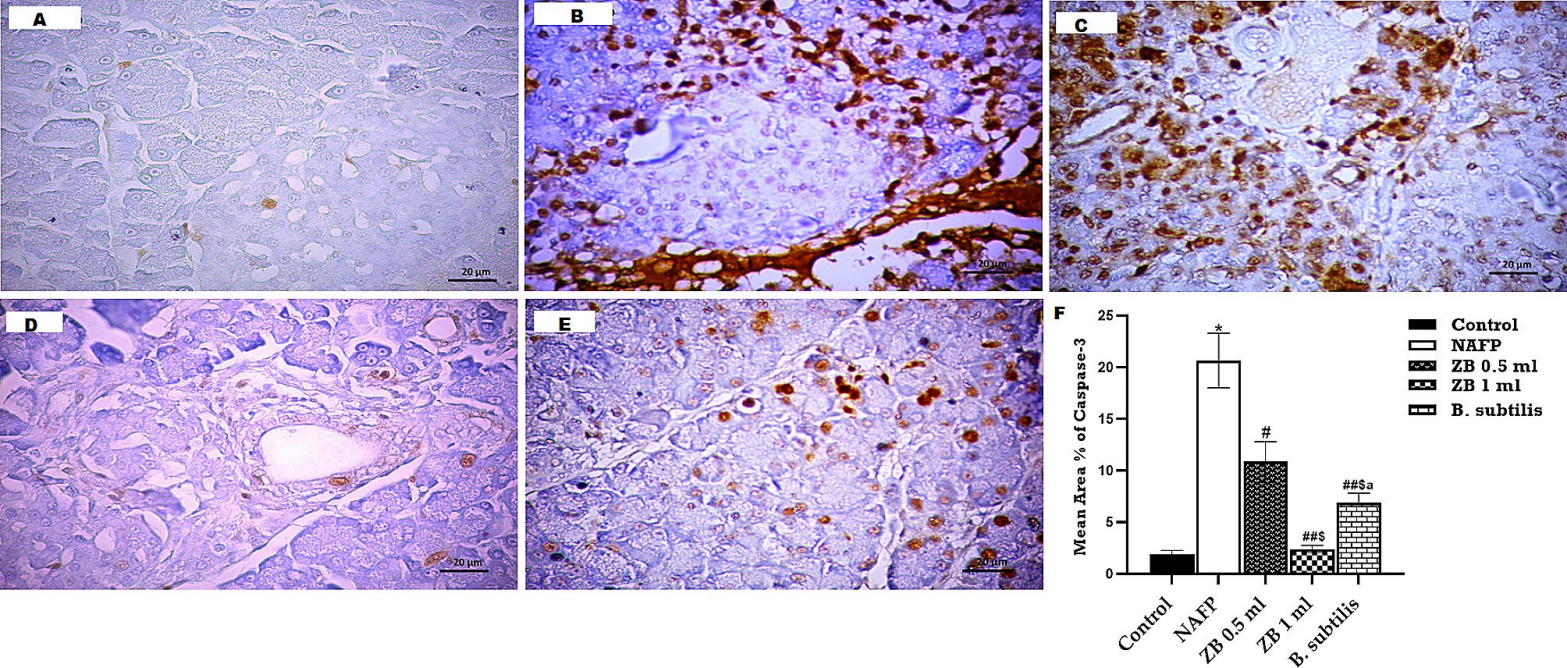
A–E Caspase-3 immunohistochemistry-stained pancreas sections of **A**: Control group reveals a minimal response of caspase-3 within the acinar cells and islet of Langerhans, **B**: NAFP model group exhibits extensive and widespread positive response among pancreatic acini and islet of Langerhans, **C**: ZB 0.5 ml group, there is still evident positive reaction for caspase-3, **D**: ZB 1 ml group revealed a minimal positive reaction for caspase-3 among pancreatic acini and islet of Langerhans. **E**: ***B***. *subtilis* group, there is moderate positive reaction for caspase 3. **F**: morphometric and statistical analysis of the area percentage of Caspase 3-positive cells. One-way analysis of variance and Tukey’s test yielded a mean ± SD for the data. *n* = 6 rats per group^*^p < 0.01 in relation to control, ^##^*p* < 0.001 & ^#^*p* < 0.05 in relation to NAFP group, ^$^*p* < 0.05 in relation to ZB 0.5 ml group and ^a^*p*<0.05 in relation to ZB 1 ml group

The immunohistochemistry analysis for Caspase-3 and NF-κB was conducted to investigate the effect of ZBiotic on pancreatic cell death and the TNF-signaling pathway, respectively. Sections stained by Caspase-3 exhibited a minimal reaction in both acinar cells and islet of Langerhans cells in the ZB 1 ml group. However, a moderate reaction was noted in the islet of Langerhans, the cytoplasm of acinar cells, and the surrounding structures in both the ZB 0.5 ml and *B. subtilis* groups, although the impact was more pronounced in the latter. Positive reactions were notably present in most acinar cells and islet of Langerhans cell in the model group (Fig. 4).

**Fig. 5:**
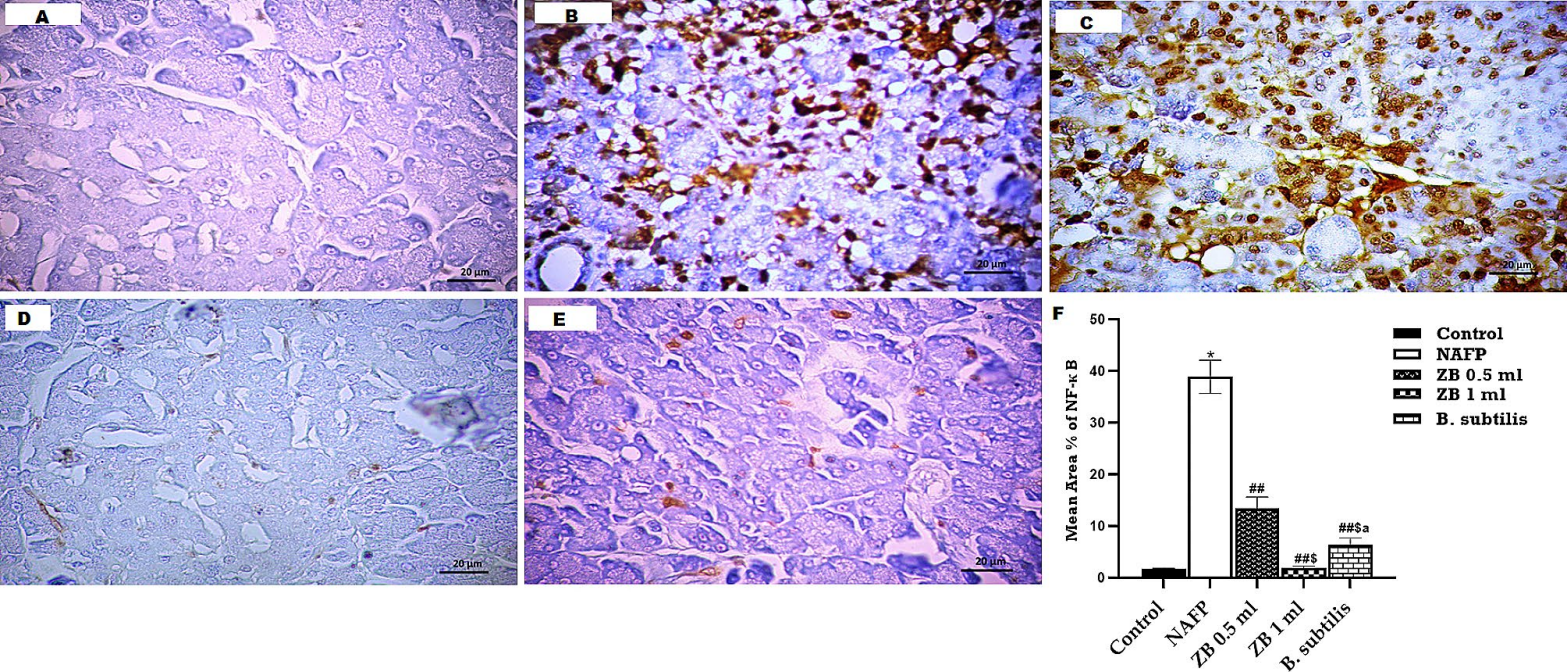
A–E NFκB1 immunohistochemistry-stained pancreas sections of A: Control group reveals a minimal reactivity for the nuclear factor kappa among pancreatic acinar cells and islet of Langerhans, B: In NAFP model group, there is a notable and widespread positive reaction for nuclear factor kappa, C: In ZB 0.5 ml group, Reaction for nuclear factor kappa in destructed areas were easily detected, D: ZB 1 ml group reveals minimal for reaction of nuclear factor kappa among pancreatic acini and islet of Langerhans. E: *B. subtilis* group, a significant decrease in reaction of nuclear factor kappa was evident in comparison to the Zbiotic treated groups and NAFP model group. **G**: morphometric and statistical analysis of the area percentage of Nuclear Factor Kappa-positive cells. One-way analysis of variance and Tukey’s test yielded a mean ± SD for the data. *n* = 6 rats per group*p < 0.01 in relation to control, ^##^*p* < 0.001 in relation to NAFP group, ^$^*p* < 0.05 in relation to ZB 0.5 ml group and ^a^*p*<0.05 in relation to ZB 1 ml group

Sections stained by NF-κB revealed a minimal reaction in acinar cells in ZB 1 ml group. However, a moderate reaction was observed in both acinar cells and islet of Langerhans in ZB 0.5 ml group. The maximum positive reaction was distinguished in most cells in the NAFP model group. Furthermore, NF-κB reactions were readily detected in the destructed areas in sections of the *B. subtilis* group. Nevertheless, the observed effect was less prominent compared to that in the ZB 0.5 ml group. A morphometric and statistical analysis of the area percentage of NF-κB-stained cells demonstrated a significant elevation in the model group compared to the other experimental groups (Fig. 5).

### Selection of the mRNAs-miRNAs panel

Bioinformatics analysis was conducted at two levels to retrieve the candidate mRNAs-miRNAs Panel associated with NAFP pathogenesis. Initially, the focus was on identifying mRNA species, specifically differentially expressed genes (DEGs), implicated in the inflammatory TNF pathway contributing to NAFP development. Subsequently, the analysis extended to identifying miRNA species targeting the identified candidate mRNAs.

### Screening for DEGs and functional enrichment analyses

By analysis of the microarray dataset, a total of 11,788 DEGs had been located in GSE194331 according to the cut-off standards (Supplementary Table S3), (Figure. S2). 5354 GO biological process terms and 320 KEGG pathways were obtained (Supplementary Table S4) after the functional enrichment analysis of the DEGs. Consequently, the functional analysis was filtered focusing on the gene sets related to necroptosis and the TNF-Mediated signaling pathway (Table S5).

### PPI Network construction and analysis and selection of candidate genes

The PPI network for necroptosis/TNF-Mediated signaling pathway-related genes was built using the STRING tool (Figure. S3A). The PPI network had 1538 edges and 146 nodes with a highly significant p-value for PPI enrichment < 1.0e-16. The betweenness indices, closeness, and degree were used to analyze the topological properties of the filtered enriched genes, and the nodes with a degree > 25 were set as hub genes (Supplementary Table S6). ZBP1 and HSPA1B from the hundred hub genes, and the additional two genes, TNF, and MAPK3 were selected for targeted co-regulatory network construction as were further validated either by several public databases or reviews [36–41] to be linked to necroptosis/TNF-Mediated signaling pathway and considered crucial insults in pancreatic injury diseases (Supplementary Figure S4& S5). Additionally, the PPI between four candidate genes was analyzed and presented a combined score > 0.3, indicating a significant confidence level. (Figure. S3B).

### Selection of candidate miRNAs

The results indicated that miR-5192 (Figure. S6) targets the four selected TNF, ZBP1, HSPA1B, and MAPK3 genes with a score of ˃0.9 (Figure. S7). Further analysis with DIANA tools mirPath revealed that miR-5192 is related to pancreatic function and disease-regulating pathways (Figure. S8).

### The effect of ZBiotic on the pancreatic selected RNA species’ expression

**Fig. 6:**
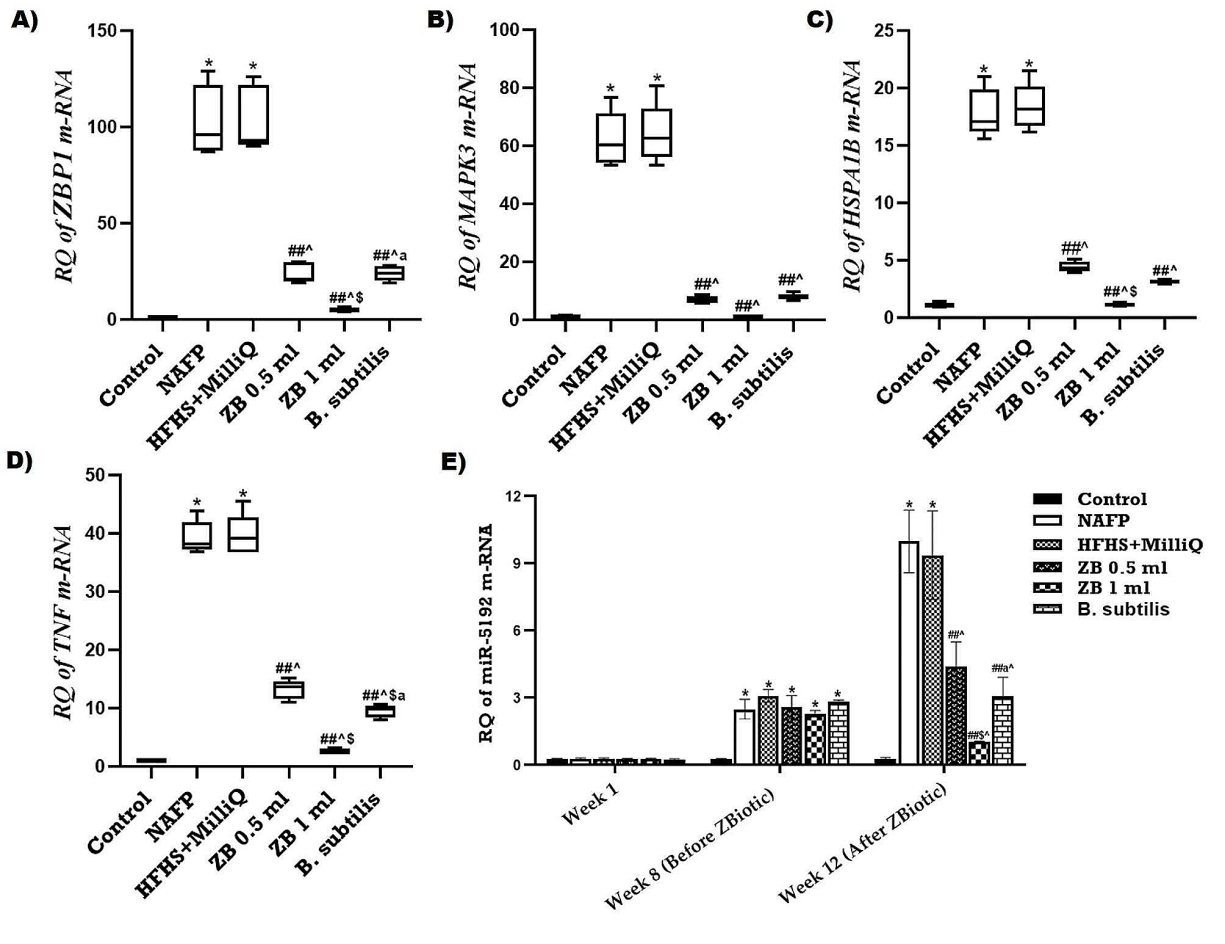
Impact of Zbiotic therapy on the expression of the pancreatic selected RNA **(A)** ZBP1, **(B)** MAPK3, (C) HSPA1B, (D) TNF, (E) miR-5192. One-way analysis of variance and Tukey’s test yielded a mean ± SD for the data. *n* = 6 rats per group*p < 0.01 in relation to control, ^##^*p* < 0.001 in relation to NAFP group, ^^^*p* < 0.05 in relation to HFHS + MilliQ group ^$^*p* < 0.05 in relation to ZB 0.5 ml group and ^a^*p*<0.05 in relation to ZB 1 ml group

The quantitative polymerase chain reaction (qPCR) assay was performed to determine the effect of ZBiotic on the expression profile of the retrieved mRNAs-miRNAs panel in the pancreatic tissue. After 8 weeks of HFHS diet manipulation, the expression of upstream miR-5192 was highly increased compared to the Control animals. Both the untreated NAFP and HFHS + MilliQ groups continued to exhibit a notable increase in the expression level of miR-5192 in comparison to the Control group, while the results also revealed a significant elevation in the expression of pancreatic TNF, ZBP1, HSPA1B, and MAPK3 mRNAs. In addition, ZBiotic administration at its two dosages (0.5 and 1 ml) significantly decreased the NAFP group animals’ untreated upregulation in pancreatic mRNA signatures expression. Furthermore, the data showed that the treated groups (0.5 and 1 ml) had significantly lower expression of miR-5192 than the untreated NAFP group (Fig. 6). In the case of the S. subtilis group, the findings indicated that the original S. subtilis stains exhibited an intermediate effect between the ZB 0.5 ml and ZB 1 ml groups in downregulating the expression of the investigated mRNA-miRNA panel, as compared to the NAFP group. Furthermore, there wasn’t any significant changes in the expression of the investigated RNAs between NAFP rats and HFHS + MilliQ animals.

## Discussion

Nonalcoholic fatty pancreatitis (NAFP) is increasingly prevalent worldwide [42] and knowing the pathogenic mechanisms underlying NAFP serves as the foundation for identifying risk factors that contribute to the progression of the disease and for finding novel therapeutic targets [43].

Emerging evidence suggests that necroptosis and the related TNF pathway are the primary drivers of Fibrogenesis and acute pancreatitis in the course of NAFP [44].

Using in silico data analysis, we created an mRNA-miRNA panel (ZBP1, HSPA1B, MAPK3 & mir-5192) associated with pancreatic cell dysfunction and metabolic syndrome and involved in necroptosis-related TNF pathway. Next, we assessed how ZBiotic treatment might modulate NAFP and how that might affect the RNA panel in the NAFP animal model.

Adipocyte infiltration into pancreatic tissue is the main mechanism responsible for explaining the incidence of fatty pancreas. Given that obesity and gaining weight are the main causes of this disease [9].

Disorders brought on by lipid infiltration impair metabolic processes in addition to morphology. β-cell exposure to free fatty acids has been shown to increase insulin release, while chronic exposure to high FFA levels induces insulin hypersecretion and β-cell hypertrophy, ultimately resulting in dysfunction and death of β-cells [45, 46].

Consequently, all previously discussed can be used to demonstrate the outcomes of the current study. As an experimental NAFP animal model, we used a high-fat and high-sucrose (HFHS) diet. A growing body of research has examined the effects of the HFHS diet on laboratory rats, and the results indicate that this diet causes insulin resistance and obesity [9, 47–49]. Furthermore, it was discovered that the hyper lipidic model was successful in inducing comorbidities related to inflammation and oxidative stress [50].

The nutritional model in this study effectively reproduced the spectrum of pathological and metabolic abnormalities linked to NAFP. The HFHS diet feeding caused elevated body weight, hyperinsulinemia, insulin resistance, hyperglycemia, and dyslipidemia in the untreated NAFP group. Along with intense mononuclear cellular infiltration, large areas of fat cells with obvious necrosis in the fat were observed in the pancreatic sections. Elevated blood levels of lipase and amylase were also observed in the animals, indicative of pancreatic damage.

Z-DNA-binding protein (ZBP1) mediates RIPK3-dependent necroptosis cell death [51] and causes pancreatic cell injury [44]. Necroptosis, a recently discovered mode of cell death, has slowly been revealed in inflammatory diseases [44]. Furthermore, recent research identified ZBP1, an IFN-inducing protein, as a key upstream regulator of proinflammatory signaling [52].

The current findings align with previously reported data, which showed that the NAFP group significantly overexpressed genes related to the pancreatic necroptosis/TNF-mediated signaling pathway (TNF, ZBP1, HSPA1B, and MAPK3) in comparison to the control group [53, 54]. These results are corroborated by immunohistochemistry and histology assays, which demonstrated the highest positive immunostaining reaction for the NFκB1 marker, along with noticeable positivity for Caspase-3, found in acinar cells, and intense mononuclear cellular infiltration in pancreatic tissue of FP group, relative to the control group. Additionally, concurrent research has confirmed that pancreatic cell injury is associated with an upregulation of NFκB1 [55, 56].

However, it has been discovered that TNF pathway, which causes apoptosis, can also cause necroptosis under specific circumstances [57]. Caspases, NF-B, and MAP kinases are among the main pathways activated by TNF [58].

It has been noted that observed that inflammatory responses and apoptosis contribute to increased caspase-3 expression. Because caspase-3 can cleave and activate cytokines to contribute to inflammatory responses [59]. Additionally, it has been noted that the animal fed a high-fat diet exhibited elevated levels of caspase-3, which was linked to notable rise in the level of inflammatory cytokines in the liver. This suggests that enhanced apoptosis could be an aggravating factor in inflammation of the liver [60]. Our findings consistently showed that the caspase-positive cell area percentage within the pancreas of NAFP model rats was noticeably greater than that of the control group, both morphometrically and statistically.

Via daily Zbiotic treatment for four weeks, among the NAFP rats that were not given treatment, all noticed disruptions were significantly alleviated. Moreover, the ZB 1 ml group demonstrated a superior effect over the original *B. subtilis* strain group in ameliorating these disturbances. At ZBiotic, researchers used homologous recombination to create new strains of *B. subtilis* ZB183TM probiotic bacteria by transferring a trait for acetaldehyde breakdown from the liver. Acetaldehyde is an alcohol metabolite that can potentially cause considerable damage, especially to the liver, pancreas, and brain. However, ZBiotic, the first genetically engineered probiotic in the world, can break down this metabolite [61].

The key enzyme responsible for acetaldehyde metabolism in the liver is acetaldehyde dehydrogenase 2 (ALDH2). ALDH is involved in various biological processes along with acetaldehyde molecule conversion. Because it has anticancer properties and can act as ‘aldehyde scavenger’ during lipid peroxidation [62]. The role of ALDH expression in NAFLD, liver fibrosis and liver cancer has been the subject of numerous investigations [63].

According to a study assessing ZBiotic toxicity, When Wistar animals were given lyophilized *B. subtilis* ZB183 spores orally via gavage for 90 consecutive days at doses of 10^9^, 10^10^, and 10^11^ CFU/kg body weight/day, there was no effect on the rats’ body weights, food consumption, ophthalmological examinations, mortality, or functional observational battery in both sexes [20].

The current study’s findings were consistent with previously available publications. The findings indicated that ZBiotic on a daily basis slowed the NAFP progression. It reduced body weight significantly, lowered insulin and blood sugar levels, improved insulin resistance, and decreased serum amylase and lipase levels.

Remarkably, both ZBiotic groups (ZB 0.5 and ZB 1) exhibited significant reductions in biochemical variables compared to the model group, indicating ZBiotic’s ability to halt pancreatic exocrine injury and reverse initial pathological changes induced by HFHS diet. Additionally, treatment reduced the expression of genes related to the necroptosis/TNF-mediated signaling pathway (TNF, ZBP1, HSPA1B, and MAPK3), as well as Caspase area percentage and positive cells for nuclear factor kappa, compared to untreated NAFP animals. In contrast to the model group, the treated groups (ZB 0.5 group) had a typical structure featuring slender interlobular septa and closely spaced pancreatic acini, as well as a significant reduction in collagen fiber percentage area. The findings suggest that ZBiotic could reduce pancreatic tissue injury by modulating the necroptosis/TNF-mediated pathway.

MiRNAs dysregulation may impact various tissue status and functions, such as the liver [28, 64, 65] and pancreas [66–68]. This dysregulation may also contribute to metabolic problems linked to disorders including obesity and insulin resistance, such as NAFP [12].

The miRWalk database identified miR-5192, with a score of > 0.9, as an upstream regulator targeting TNF, ZBP1, HSPA1B, and MAPK3 mRNAs. Further analysis with DIANA tools mirPath revealed miR-5192’s association with pancreatic function and disease-regulating pathways. Previous studies have linked miR-5192 to cancer development [69]. Surprisingly, miR-5192’s functional enrichment analysis revealed that it is strongly linked to necroptosis and related TNF signaling pathways. MiR-5192 may regulate the activity level of these genes by directly or indirectly activating gene expression in response to different types of cells and circumstances [70].

As a consequence, the results indicated a notable rise in the pancreatic miR-5192 expression level among those with untreated NAFP as opposed to the control. In comparison to the NAFP group, ZBiotic administration significantly reduced its expression.

**Fig. 7:**
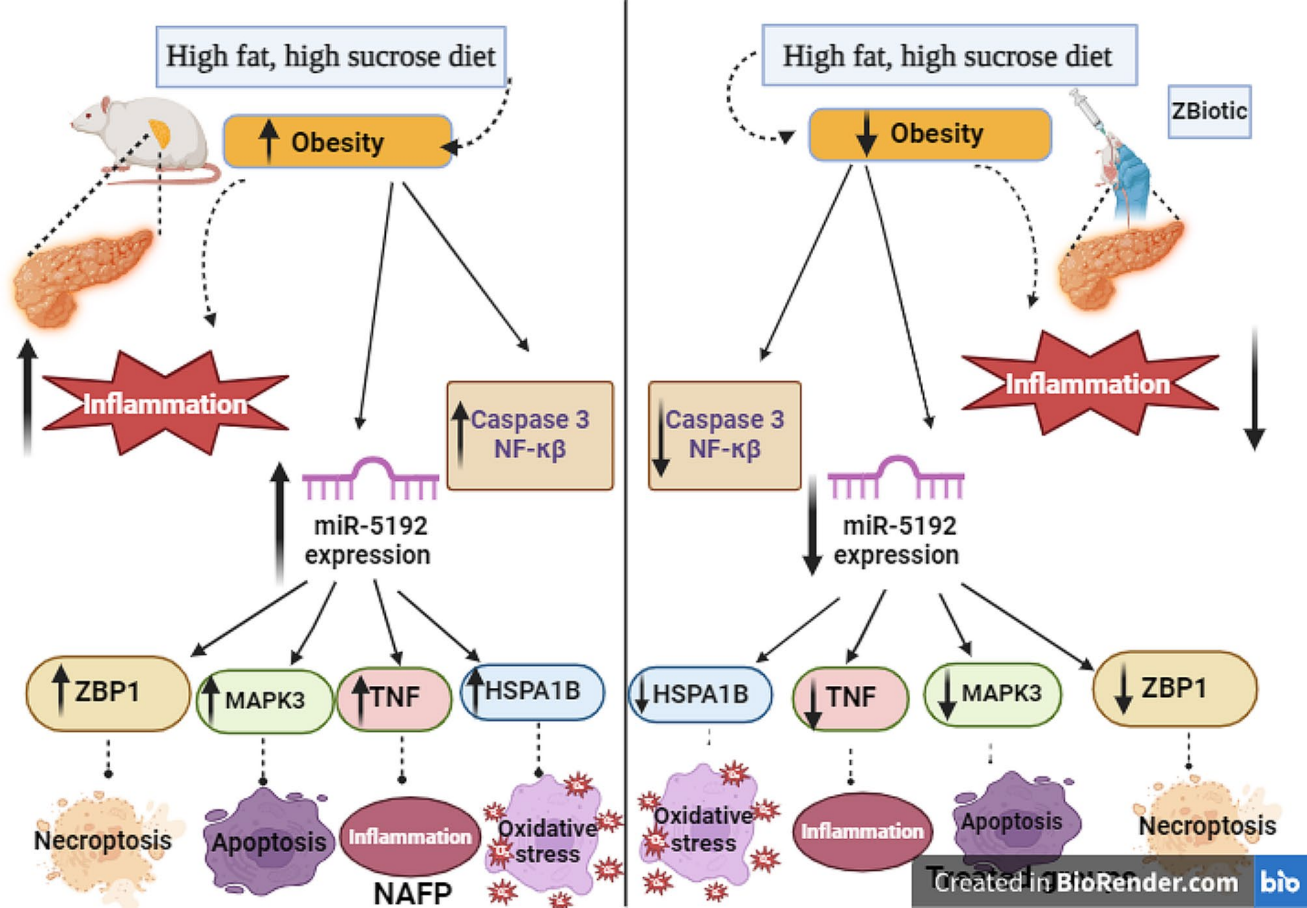
Schematic representation and summary and of the study hypothesis

We postulated that (Fig. 7) pancreatic TNF, ZBP1, HSPA1B, and MAPK3 were all upregulated as a result of miR-5192 being expressed more frequently in HFHS-induced lipotoxicity (untreated NAFP). In addition to stimulating several downstream signaling pathways and promoting the expression of inflammatory responses (NFκB1 and caspase 3), activating the associated TNF signaling pathway and necroptosis also increased the fibrosis and elevated the levels of serum lipase and amylase. As a result, pancreatic cell damage increase. The current study may contribute to enhanced comprehension of the pathophysiology and etiology of NAFP, along with helpful knowledge regarding possible genetic targets for NAFP therapy. Nevertheless, until more preclinical research is done, ZBiotic—an example of genetically modified bacteria—might not be an appropriate basic therapy approach.

However, the study is subject to certain limitations, notably the necessity for additional mechanistic investigations to explore the potential effects of Zbiotic on oxidative stress markers, as well as the examination of protein levels of the constructed TNF pathway/necroptosis-related RNA panel. Furthermore, there is a need for further implementation of comparative studies. The authors are forging ahead with 16s rRNA gene sequencing of stool samples to uncover precise *B. subtilis* ZB183 strain-induced transformations in gut microbiota diversity and abundance across the experimental groups.

## Conclusion

ZBiotic has shown promise in reducing the development of NAFP, preventing fibrosis and inflammation of pancreatic cells, as well as lessening the metabolic and pathological abnormalities observed in the used NAFP animal model. Moreover, the Zbiotic in its high dose proved a superior effect over the original *B. subtilis* strain in ameliorating these disturbances. The observed impact was related to decreased expression of miR-5192 and decreased expression of pancreatic TNF, ZBP1, HSPA1B, and MAPK3 mRNAs (Fig. 7).

### Electronic supplementary material

Below is the link to the electronic supplementary material.

Supplementary Material 1

## Data Availability

No datasets were generated or analysed during the current study.
